# Association of ABO Blood Types and Clinicopathological Features of Prostate Cancer

**DOI:** 10.1155/2017/9237481

**Published:** 2017-10-09

**Authors:** Fang-Ming Wang, Yan Zhang, Gui-Ming Zhang, Ya-Nan Liu, Li-Jiang Sun, Yong Liu

**Affiliations:** ^1^Department of Urology, The Affiliated Hospital of Qingdao University, Qingdao, Shandong 266003, China; ^2^Division of Dyslipidemia, State Key Laboratory of Cardiovascular Disease, Fu Wai Hospital, National Center for Cardiovascular Diseases, Chinese Academy of Medical Sciences, Peking Union Medical College, Bei Li Shi Road 167, Beijing 100037, China

## Abstract

**Purpose:**

To investigate the association between ABO blood types and clinicopathological characteristics in patients with prostate cancer (PC).

**Methods:**

A total of 237 pathologically diagnosed PC patients were enrolled. All patients were classified as low–middle or high-risk group. The correlation of ABO blood types with high-risk PC was determined by univariate and multivariate regression analysis.

**Results:**

Data indicated 144 (85.7%) patients were stratified as high risk in the non-O group, while 50 (72.5%) patients in the O group (*p* = 0.025). However, there was no significant difference regarding PSA, Gleason score, stage, or metastasis between O and non-O group (*p* > 0.05). Univariate logistic regression analyses revealed PSA, Gleason score, and blood type non-O were all correlated with high-risk PC (OR = 1.139, *p* < 0.001; OR = 9.465, *p* < 0.001; OR = 2.280, *p* = 0.018, resp.). In the stepwise multivariate regression analysis, the association between blood type non-O and high-risk PC remained significant (OR = 33.066, 95% CI 2.391–457.323, and *p* = 0.009) after adjusting for confounding factors as well as PSA and Gleason score.

**Conclusion:**

The present study firstly demonstrated that non-O blood type was at higher risk of aggressive PC compared with O type, suggesting that PC patients with non-O blood type should receive more attention in clinical practice.

## 1. Introduction

Prostate cancer (PC) is the most common cancer among males in the Western world, with more than 1.11 million new cases diagnosed in 2012 and 307,000 deaths [1, 2]. PC is also now becoming an emerging health priority in East Asia due to exposure to westernized diet and lifestyle [3]. It is expected that the incidence will substantially increase in the coming decades due to the aging population, which makes it a huge health care problem. It is crucial and desirable to evaluate and predict the risk of PC appropriately.

Although preoperative prostate-specific antigen (PSA) levels, Gleason score in the biopsy specimen, and clinical stages are well-established predictors of PC, identification of additional factors which can help us to better estimate and evaluate the severity of PC is expected to be useful for patient counseling. The ABO blood group is determined by the presence of A or B blood group antigens on the surface of red blood cells, which consist of proteins and carbohydrates attached to lipids or proteins. ABO blood group antigens are also found in a variety of epithelial cells; therefore, the clinical significance of the ABO blood group system extends beyond transfusion medicine [4]. The ABO blood group has been associated with a number of nonneoplastic diseases [5–10]. Furthermore, associations have also been made between the ABO blood group and certain malignancies including cancers of the pancreas [11, 12], ovary [13], kidney [14], and skin [15]. For example, it has been reported that individuals with non-O blood groups have an elevated risk of developing gastric and pancreatic cancers [4].

To date, few studies have investigated potential associations between ABO blood groups and the risk of PC. Lack of association between blood type and PC risk has been reported [12]. However, to the best of our knowledge, whether ABO blood types associated with the clinicopathological characteristics of pathologically confirmed PC has not been reported. Accordingly, in the present study, we sought to determine the association between ABO blood groups and clinicopathological features including the risk of PC in 237 pathologically diagnosed patients.

## 2. Methods

### 2.1. Study Design and Population

The study complied with the Declaration of Helsinki and was approved by our Institute Ethical Committee. All subject names, initials, or hospital numbers were not used in the text, table, or illustrative materials of this study.

The study was conducted in patients with primary diagnosed, pathologically confirmed sporadic PC, between January 2011 and August 2016 at the Department of Urology at the Affiliated Hospital of Qingdao University. The exclusion criteria of the study were the presence of medical history of other malignancies, incomplete information of pathological characteristics, or ABO blood type records. All data on age, body mass index (BMI), history of hypertension or diabetes, serum PSA, biopsy cancer grade (Gleason score), tumor clinical stage at diagnosis, treatment protocols, and ABO blood type status were obtained from electronic records and medical charts. All the pathological data analyzed in this study were identified from the transrectal ultrasound-guided prostate biopsy specimens. All specimens were processed according to standard pathological procedures. Tumor stage was assessed according to the American Joint Committee on Cancer (AJCC) TNM classification of malignant tumors 2002. Gleason score was assessed according to the ISUP classification of 2005 [16].

### 2.2. Exposure Definition

Risk factor definitions were as follows. (1) BMI was defined as the first reported weight (in kilograms) divided by height in square meters, and BMI ≥ 25 kg/m^2^ was considered as overweight; (2) the threshold of hypertension was set at 140 and 90 mmHg for systolic and diastolic blood pressure, respectively, on three consecutive occasions; and (3) diabetes was based on either one of the following criteria: fasting serum glucose level ≥ 7.0 mmol/L, normal fasting serum glucose level owing to usage of antidiabetic medication, or self-report of a physician's diagnosis of diabetes.

### 2.3. Statistical Analyses

Quantitative variables were expressed as mean ± standard deviation (SD) and were analyzed by the Student's *t*-tests or one way ANOVA as appropriate. The qualitative variables were expressed as numbers and percentages and were analyzed by chi-squared statistic tests. Correlations between variables with high-risk PC were examined by univariate logistic regression analysis. Stepwise multivariate regression analysis was used to determine the independent factors of high-risk PC. A *P* value of less than 0.05 was considered statistically significant. Statistical studies were carried out with the SPSS program (version 19.0, SPSS, Chicago, Illinois, USA).

## 3. Results

### 3.1. Baseline Characteristics

The current study consisted of 237 eligible pathologically confirmed PC patients (male, with a mean age of 70.7 ± 7.9 years). The mean Gleason score was 8.1 ± 1.3. The clinical stage was as follow: T1 (12 cases), T2a (31 cases), T2b (50 cases), T2c (34 cases), T3 (77 cases), and T4 (33 cases). The prevalence of the various blood groups in this study was as follows: O, 29.1% (*n* = 69); A, 27.8% (*n* = 66); B, 33.3% (*n* = 79); and AB, 9.7% (*n* = 23). The distribution of ABO blood groups was generally similar to that found in the general population of Han Chinese [17]. The baseline demographic, clinical characteristics, and laboratory findings of the enrolled subjects were summarized in Table 1.

All PC subjects were divided into two groups: low–middle risk group (*n* = 43) and high-risk group (*n* = 194) according to PSA, Gleason score, and clinical stage. Low–middle risk is defined as follows: PSA ≤ 20 ng/ml, Gleason Score ≤ 7, and clinical stage ≤ T2b; high risk was based on either one of the following criteria: PSA > 20 ng/ml, Gleason Score ≥ 8, or clinical stage ≥ T2c.

Although there was no significant association between different risk groups and the ABO blood types (*p* = 0.086), the trend was evident that patients with O blood type tended not to have high risk (high-risk percentage: O versus A versus B versus AB: 72.5% versus 86.4% versus 83.5% versus 91.3%). Then, we further evaluated the relation of O and non-O subgroups to the PC risks and finally found that blood type O patients had a significantly lower risk of aggressive prostate cancer compared to type non-O patients (high-risk percentage: O versus non-O: 72.5% versus 85.7%, *p* = 0.025) (Table 1).

To evaluate the clinicopathological characteristics according to ABO blood types, as shown in Table 2, we observed that 144 (85.7%) patients were stratified as high risk in the non-O group, while 50 (72.5%) patients were stratified as high risk in the O group (*p* = 0.025). However, there was no significant difference between non-O and O group, regarding PSA, Gleason score, clinical stage, lymph node involvement, or bone metastasis (*p* = 0.999, 0.234; *p* = 0.434; *p* = 0.232; *p* = 0.542; and *p* = 0.558, resp.).

### 3.2. Correlations of ABO Blood Types and Other Clinical Variables with High-Risk PC

To evaluate the correlations of ABO blood types and other clinical variables with high-risk PC, univariate logistic regression analysis was performed in the current study. As shown in Table 3, parameters including PSA, Gleason score, and blood type O or non-O found to be statistically significant in univariate analyses were entered into multivariate logistic regression analysis. The data indicated that PSA, Gleason score, blood type O, and non-O were independently correlated with the presence of high-risk prostate cancer in multivariate logistic regression analysis. In particular, non-O blood type was proved to be an independent predictor for the presence of high-risk PC after adjusting for the known confounders (OR = 2.28, 95% CI 1.152–4.512, and *p* = 0.018) (Table 4). Nevertheless, we did not observe statistically significant relationship between high-risk PC and age, BMI, hypertension, diabetes mellitus, coronary artery disease, or other blood subgroups (Table 3).

## 4. Discussion

The present study evaluated the association of ABO blood types and clinicopathological features including the risk of PC. To our knowledge, this is the first study to demonstrate that patients with O blood type tended to have lower risk and to be less aggressive compared with non-O blood types. This association was independent of PC risk factors, since it remained significant after adjusting for other confounders. Therefore, in addition to PSA, clinical stage, and Gleason score, the current study might provide novel information with regard to the assessment of PC risk from the clinical perspectives.

ABO antigens are highly expressed not only in red blood cells but also in human tissues and most epithelial and endothelial cells [18, 19]. Red blood cell antigens have various functions, such as membrane structural integrity, transportation of molecules through membranes, and adhesion [20]. Since ABO blood types were found to be associated with gastric cancer in 1953 by Arid et al. [21], the relationship between the ABO blood group and the risk, incidence, and clinicopathologic characteristics of human tumors has been suspected. Previous studies have generally shown an increased risk of cancer, including pancreas, ovarian, and renal, for non-type O blood group compared to type O [13, 14, 22]. Wolpin et al. reported that people with non-O blood groups have an adjusted hazard ratio (HR) for pancreatic cancer of 1.44 (95% confidence interval [CI] 1.14–1.82), compared with people with blood group O [22]. Several plausible mechanisms, such as intercellular adhesion and membrane signaling, inflammation, and immune surveillance of malignant cells, have been proposed to explain this observed association between the ABO blood group and cancer risk [22]. Moreover, some studies have suggested a possible relationship between ABO blood group antigens and progression of human tumors [15, 23–27].

However, few studies on the relationship between ABO blood types and PC have been reported. We reviewed the literatures and found that Rummel and Ellsworth [12] had reported that there was no association between blood type and PC risk or survival, but whether loss of antigen expression increases with tumor progression or invasion is controversial. Besides, one recent study demonstrated that there is no association between ABO blood type and risk of aggressive PC [28]. Almost all the mentioned studies compared the PC patients with normal people and did not reveal the relationship between ABO blood types and risk of aggressiveness or invasion in patients with PC.

Our study divided PC patients into two groups: Low–middle-risk group and high-risk group, and then analyzed the association of ABO blood types and clinicopathological features in the two groups. Our study showed, by both univariate and multivariate analyses, blood type non-O were independently correlated with the presence of high-risk PC. Compared with patients with other ABO blood groups, patients with blood group O had significantly lower risk of invasion. Although, there were no significant differences on bone metastasis or positive pelvic lymph node between individuals with different ABO blood types, we can also see a trend that patients with blood group O had significantly lower risk of bone and lymph node metastasis, which was consistent with the main result of our study.

Although the study design is different, our findings are similar to those of previous studies [12] in other cancer fields including pancreatic, gastric, breast, colorectal, ovarian, and esophageal cancer, all of which revealed that blood group O decreased cancer risk compared with the non-O group. Moreover, our present study provided that in PC patients, non-O blood type was strongly and positively associated with high risk, which was independent of other confounders including the established PC risk factors such as PSA and Gleason score. Therefore, our data extended previous studies and provided new information with regard to the clinical application of ABO blood types.

Given the potential mechanisms of this correlation of ABO blood types and clinicopathological features remain uncertain and need further studies, we suggest PC patients with non-O blood type may have higher risk and should receive more attention in clinical practice. As for the treatment protocols, the percentage of radical prostatectomy in low–middle-risk group was significantly higher than that in the high-risk group because some patients with high risk especially with metastasis lost the opportunity of radical prostatectomy when diagnosis was confirmed.

There were several limitations in our study. Firstly, it was conducted at a single center. Secondly, this was an observational study and so still there could be residual confounding. Thirdly, we did not evaluate the prognostic value of ABO blood type in our population. Finally, we did not apply the new 2014 ISUP grading system to our study due to pathological report limitations. Further study is needed to examine the role of ABO blood type in predicting the clinical outcomes in a large sample size and long-term follow-up.

## 5. Conclusions

In conclusion, this study provides evidence of an association between the ABO blood group and clinicopathological features of prostate cancer: patients with blood group O had lower risk rate than patients with non-O blood groups. This association remained significant after adjustment for other known risk factors. Further basic research on tumor genetic or biological differences associated with the ABO blood group is needed in the future studies.

## Figures and Tables

**Table 1:** Baseline characteristics of the study population according to the risk of PC.

Variables	All subjects (*n* = 237)	Low–middle risk (*n* = 43)	High risk (*n* = 194)	*P* value
*Demographic Characteristics*				
Age (years)	70.7 ± 7.9	70.1 ± 6.6	70.8 ± 8.2	0.61
BMI (*𝑛* (%))				0.231
<25	138 (58.2)	29 (67.4)	109 (56.2)	
≥25	99 (41.8)	14 (32.6)	85 (43.8)	
Hypertension (*𝑛* (%))	85 (35.9)	13 (30.2)	72 (37.1)	0.483
Diabetes mellitus (*𝑛* (%))	37 (15.6)	7 (16.3)	30 (15.5)	0.894
Coronary artery disease (*𝑛* (%))	38 (16.0)	5 (11.6)	33 (17.0)	0.494
*Clinicopathological characteristics*				
PSA (ng/ml)				
PSA ≤ 100 (ng/ml)	30.4 ± 26.3	10.4 ± 4.9	38.6 ± 27.1	**<0.001**
PSA > 100 (*𝑛* (%))	87 (36.7)	0 (0)	87 (45.5)	**<0.001**
Gleason score	8.1 ± 1.3	6.4 ± 0.5	8.5 ± 1.1	**<0.001**
Stage				**<0.001**
T1	12 (5.1)	10 (23.3)	2 (1.0)	
T2a	31 (13.1)	15 (34.9)	16 (8.2)	
T2b	50 (21.1)	18 (41.9)	32 (16.5)	
T2c	34 (14.3)	0 (0)	34 (17.5)	
T3	77 (32.5)	0 (0)	77 (39.7)	
T4	33 (13.9)	0 (0)	33 (17.0)	
*N*				**<0.001**
N0	160 (67.5)	43 (100)	117 (60.3)	
N1	77 (32.5)	0 (0)	77 (39.7)	
*M*				**<0.001**
M0	147 (62.0)	43 (100)	104 (53.6)	
M1	90 (38)	0 (0)	90 (46.4)	
*ABO type, n (%)*				0.086
O	69 (29.1)	19 (27.5)	50 (72.5)	
A	66 (27.8)	9 (13.6)	57 (86.4)	
B	79 (33.3)	13 (16.5)	66 (83.5)	
AB	23 (9.7)	2 (8.7)	21 (91.3)	
*O/non-O group, n (%)*				**0.025**
O	69 (29.1)	19 (27.5)	50 (72.5)	
non-O	168 (70.9)	24 (14.3)	144 (85.7)	
*Surgery, n (%)*				
Radical prostatectomy	47 (19.8)	19 (44.2)	28 (14.4)	**<0.001**
Bilateral orchiectomy	56 (23.6)	0 (0)	56 (28.9)	**<0.001**

Data are expressed as *n* (%) or mean ± SD. PC = prostate cancer; BMI = body mass index; PSA = prostate-specific antigen.

**Table 2:** Pathological features of the study population stratified by ABO blood type.

Variables	ABO blood types				non-O (*n* = 168)	*P* value^a^	*P* value^b^
	O (*n* = 69)	A (*n* = 66)	B (*n* = 79)	AB (*n* = 23)			
PSA							
PSA ≤ 100 (ng/ml)	30.4 ± 27.5	28.0 ± 25.4	31.0 ± 24.5	34.8 ± 31.8	30.4 ± 25.9	0.861	0.999
PSA > 100 (*𝑛* (%))	21 (30.9)	27 (40.9)	31 (40.3)	8 (34.8)	66 (39.8)	0.598	0.234
Gleason score	8.03 ± 1.4	8.29 ± 1.2	8.15 ± 1.3	8.00 ± 1.2	8.18 ± 1.2	0.099	0.434
Stage						0.350	0.232
T1	3 (4.3)	1 (1.5)	7 (8.9)	1 (4.3)	9 (5.4)		
T2a	17 (24.6)	6 (9.1)	5 (6.3)	3 (13.0)	14 (8.3)		
T2b	14 (20.3)	15 (22.7)	17 (21.5)	4 (17.4)	36 (21.4)		
T2c	8 (11.6)	9 (13.6)	12 (15.2)	5 (21.7)	26 (15.5)		
T3	16 (23.2)	27 (40.9)	28 (35.4)	6 (26.1)	61 (36.3)		
T4	11 (15.9)	8 (12.1)	10 (12.7)	4 (17.4)	22 (13.1)		
*N*						0.550	0.542
N0	49 (71)	41 (62.1)	56 (70.9)	14 (60.9)	111 (66.1)		
N1	20 (29)	25 (37.9)	23 (29.1)	9 (39.1)	57 (33.9)		
*M*						0.720	0.558
M0	45 (65.2)	38 (57.6)	51 (64.6)	13 (56.5)	102 (60.7)		
M1	24 (34.8)	28 (42.4)	28 (35.4)	10 (43.5)	66 (39.3)		
High risk	50 (72.5)	57 (86.4)	66 (83.5)	21 (91.3)	144 (85.7)	0.086	**0.025**

Data are expressed as *n* (%) or mean ± SD. The bold value indicated statistical significance. PC = prostate cancer; BMI = body mass index; PSA = prostate-specific antigen. *P* value^a^ for comparison among ABO blood types; *P* value^b^ for comparison between non-O and O blood types.

**Table 3:** Univariate analysis for high-risk patients with PC.

	Univariate model		*P* value
	OR	95% CI	
Age	1.011	0.970–1.054	0.608
BMI	1.615	0.804–3.247	0.178
Hypertension	1.362	0.668–2.778	0.396
Diabetes mellitus	0.941	0.383–2.310	0.894
Coronary artery disease	1.558	0.570–4.255	0.387
PSA	1.139	1.077–1.205	**<0.001**
Gleason score	9.465	4.869–18.400	**<0.001**
O (versus non-O)	0.439	0.222–0.868	**0.018**
A (versus non-A)	0.511	0.243–1.074	0.077
B (versus non-B)	0.659	0.331–1.311	0.235
AB (versus non-AB)	0.402	0.091–1.783	0.230
non-O (versus O)	2.280	1.152–4.512	**0.018**

Univariate regression analyses are applied. The bold value indicated statistical significance. PC = prostate cancer; BMI = body mass index; PSA = prostate-specific antigen.

**Table 4:** Multivariate analysis to identify the independent correlation between non-O blood type and high risk of PC.

Models	Variables	Multivariate model		*P* value
		OR	95% CI	
1	Non-O	2.280	1.152–4.512	**0.018**
2	Model 1 + covariates	2.443	1.215–4.914	**0.012**
3	Model 2 + PSA	3.525	1.221–10.178	**0.020**
4	Model 3 + Gleason score	33.066	2.391–457.323	**0.009**

Multivariate regression stepwise models are shown. The bold value indicated statistical significance. The dependent variable was high-risk PC. Model 1 was unadjusted. Model 2 corrected for covariates including age, body mass index, hypertension, diabetes mellitus, and coronary artery disease. Model 3 additionally corrected for PSA based on Model 2; Model 4 additionally corrected for Gleason Score based on Model 3. PSA = prostate-specific antigen; PC = prostate cancer.
